# Characterization of the therapeutic effect of antibodies targeting the Ebola glycoprotein using a novel BSL2-compliant rVSVΔG-EBOV-GP infection model

**DOI:** 10.1080/22221751.2021.1997075

**Published:** 2021-11-10

**Authors:** Ha-Na Lee, Ian L. McWilliams, Aaron P. Lewkowicz, Kaliroi Engel, Derek D. C. Ireland, Logan Kelley-Baker, Seth Thacker, Pedro Piccardo, Mohanraj Manangeeswaran, Daniela Verthelyi

**Affiliations:** a Division of Biotechnology Review and Research-III, Office of Biotechnology Products, Center for Drug Evaluation and Research, Food and Drug Administration, Silver Spring, MD, USA; b Center for Biologics Evaluation and Research, Food and Drug Administration, Silver Spring, MD, USA

**Keywords:** Ebola virus (EBOV), EBOV glycoprotein (EBOV-GP), EBOV-GP pseudotyped vesicular stomatitis virus (VSV-EBOV), BSL-2 model, CNS infection, SAB-139, meningoencephalitis

## Abstract

Ebola virus (EBOV) infections cause haemorrhagic fever, multi-organ failure and death, and survivors can experience neurological sequelae. Licensing of monoclonal antibodies targeting EBOV glycoprotein (EBOV-GP) improved its prognosis, however, this treatment is primarily effective during early stages of disease and its effectiveness in reducing neurological sequela remains unknown. Currently, the need for BSL4 containment hinders research and therapeutic development; development of an accessible BSL-2 *in vivo* mouse model would facilitate preclinical studies to screen and select therapeutics. Previously, we have shown that a subcutaneous inoculation with replicating EBOV-GP pseudotyped vesicular stomatitis virus (rVSVΔG-EBOV-GP or VSV-EBOV) in neonatal mice causes transient viremia and infection of the mid and posterior brain resulting in overt neurological symptoms and death. Here, we demonstrate that the model can be used to test therapeutics that target the EBOV-GP, by using an anti-EBOV-GP therapeutic (SAB-139) previously shown to block EBOV infection in mice and primates. We show that SAB-139 treatment decreases the severity of neurological symptoms and improves survival when administered before (1 day prior to infection) or up to 3 dpi, by which time animals have high virus titres in their brains. Improved survival was associated with reduced viral titres, microglia loss, cellular infiltration/activation, and inflammatory responses in the brain. Interestingly, SAB-139 treatment significantly reduced the severe VSV-EBOV-induced long-term neurological sequalae although convalescent mice showed modest evidence of abnormal fear responses. Together, these data suggest that the neonatal VSV-EBOV infection system can be used to facilitate assessment of therapeutics targeting EBOV-GP in the preclinical setting.

## Introduction

Ebola virus (EBOV) is a highly pathogenic member of the Filoviridae family that can cause fever, diarrhoea, dehydration, and malaise followed by systemic bleeding, multi-organ failure, shock and death [1]. In the acute phase, neurological symptoms in patients with Ebola virus disease (EVD) can range from headache, to altered mental status, seizures and coma. If untreated, mortality for EBOV ranges between 20 and 90%. Importantly, survivors report seizures, memory loss, headaches, cranial nerve abnormalities, and tremors [2–7]. Indeed, early results from the PREVAIL trial, which is following survivors of EBOV infections for 5 years, show that most survivors experience headaches, and memory loss [8]. Lastly, the virus has been recovered from cerebrospinal fluid of convalescent subjects that consistently test negative for the virus in peripheral blood, and emerging data shows that survivors may be the source of new infections because they can harbour and intermittently shed EBOV reinforcing the need to eliminate the pathogen from all tissues [9,10]. Despite a clear effect of EBOV in the central nervous system (CNS), the risks associated with EBOV infections have resulted in very limited necropsy or high resolution imaging data of the infected CNS to date, limiting our understanding of host–pathogen interactions in the CNS [11]. Together, this underscores the need to develop a model that enables a better understanding of host–pathogen interactions, learns more about the cells that are targeted by the EBOV surface glycoprotein (EBOV-GP) in the brain, and finds therapeutics capable of completely eradicating the virus from the CNS and other immune privileged sites [12].

EBOV is a negative strand RNA virus. The only protein expressed on its surface is the membrane-anchored envelope glycoprotein (GP). EBOV attachment and entry into cells is complex, and can be mediated by a number of different entry factors depending on the cell type [13–17]. Once internalized, GP interacts with NPC1, facilitating fusion and endosomal release [18]. Since the GP mediates the attachment, internalization, and endosomal release of the virus into the cell, it is the main target of most proposed and licensed therapeutics for EBOV [19,20].

Recently developed monoclonal antibodies targeting EBOV-GP, Inmazeb^TM^ and Ebanga^TM^ have improved survival significantly, but their efficacy is limited in patients with high viremia or with advanced disease [21,22]. Further, their effectiveness at reducing long-term neurological sequelae or their ability to neutralize other species of EBOV has not been established. The complexity of the EBOV tropism, pathogenicity, and potential for viral recrudescence underscores the necessity to develop a variety of medical interventions to treat EVD for multiple settings.

To date, understanding host–pathogen interactions and development and validation of EBOV therapeutics has been hampered by the time, labour and cost associated with working in biosafety level 4 (BSL-4) facilities [23]. Further, limited space in high-containment vivarium and ethical issues severely limit the use of the use of non-human primates (NHP), the preferred animals to model infection. Moreover, since immune competent mice are not susceptible to infection, current studies use severely immunodeficient mice, mouse-adapted EBOV (MA-EBOV), or humanized mice [24]. Thus, the development of an immune competent symptomatic murine model that uses EBOV Zaire’s GP to test therapeutics under BSL-2 conditions would facilitate the screen of potential EBOV therapeutics. In a recent study, we demonstrated that subcutaneous inoculation of neonatal C57BL/6 mice with replicating vesicular stomatitis virus pseudotyped with EBOV-GP (rVSVΔG-EBOV-GP; also known as VSV-EBOV) results in transient viremia, and infection of the brain and eyes. In addition, mice showed failure to gain weight, neurological symptoms, and then death 7–12 days post infection (dpi). In the brain, VSV-EBOV localizes first to the mid brain and pons and then to cerebellum, where it infects neurons in the granular and Purkinje layers, resulting in progressive foci of apoptosis and neurodegeneration [25]. The disease is EBOV-GP dependent as VSV bearing the glycoprotein from EBOV Reston did not succumb to the infection [25]. Since VSV-EBOV is amenable to BSL-2 conditions, we reasoned this model could be used to test therapeutics for EBOV that target the GP. The model would be useful to improve our understanding of host–pathogen interactions, and to assess the effect of therapeutics or combination of therapeutics targeting EBOV-GP in clearing virus from immunoprivileged sites such as the CNS. To examine whether we could use the model to test EBOV therapeutics, we used a polyclonal anti-EBOV-GP antibody, SAB-139 that was generated in transchromosomic humanized cows vaccinated with recombinant EBOV-GP nanoparticles (3 inoculation of 2 mg/kg) [26]. The resulting human polyclonal antibody was previously shown to have high binding avidity to EBOV-GP, strong virus neutralization and Fc-mediated effector function. Moreover, the polyclonal preparation was previously shown to control the infection in mice challenged with MA-EBOV as well as in NHP challenged with EBOV [26,27].

## Materials and methods

### Mice

C57BL/6J (B6) mice were purchased from Jackson Laboratory. Naïve three-day-old (P3) neonatal mice born from specific pathogen-free parents were randomly split into groups for use in this study and these mice were not separated by gender as gender assignment is difficult before day 15. Mice were bred and housed in the FDA AAALAC accredited, pathogen-free animal facility. Mice were housed in standard cages with 1 breeding pair or up to 5 single sex mice per cage and a 12/12 light/dark schedule and fed on commercial 5P76 Prolab Isopro RMH 3000 diet. The experimental protocol 2016-13 was reviewed and approved by the FDA Animal Care and Use Committee (FDA-ACUC), and all animals used in these studies conform to relevant regulatory standards. All procedures were performed in accordance with the FDA ACUC guidelines.

### Pseudotyped viruses

As described in McWilliams et al. [25], we obtained the VSV-G-deleted Vesicular Stomatitis Virus containing the Zaire (Mayinga) Ebolavirus glycoprotein (VSV-EBOV) from BEI Resources (established by NIAID and maintained by ATCC). Viral stocks were passaged in Vero E6 cells to produce a master stock and stored at –80°C. Viral quantification of stocks was performed by TCID_50_.

### Cell lines

VERO E6 kidney epithelial cell-line was purchased from ATCC (Cat. CRL-1586) and cultured in MEM media containing 10% FBS, 1% Pen-Strep, and 1% L-Glutamine in a 37°C, 5% CO_2_ incubator for the duration of culture. The sex of this cell line is not specified by ATCC.

### Mouse infections and treatments

Viral stocks were thawed before each infection and diluted to working concentration to administer 1000 TCID_50_ of virus in 50 ul sterile phosphate buffer solution (PBS) subcutaneously (s.c.) in the scruff of the neck of P3 neonatal mice. SAB-139 and control antibodies were graciously gifted to us by SAb Biotherapeutics (Sioux Falls, SD). Stocks were diluted to 10mg/ml in saline and administered intraperitoneally (i.p.) at 100 mg/kg in mice at either −1 (pretreatment), 1, or 3 dpi. Mice that were determined to be moribund per criteria pre-established in the protocol by laboratory or animal staff were euthanized and counted as dead. Weight change was calculated as the change in the mean experimental group weight over time for each experiment [(Average group weight at day X post infection)/ (Average starting weight for the same group) * 100]. Mice that were euthanized to collect tissues for IHC were immediately exsanguinated by perfusion with cold PBS.

### TCID_50_ viral quantification

VERO E6 cells were plated in 100ul of complete MEM media in a 96 well plate to a target confluency of 70-90% at 24 h after plating. Organs were homogenized and cleared of cellular debris by centrifugation. The cleared supernatant was then serial diluted in non-supplemented MEM media and 100ul/well was plated in the VERO E6 96 well plate. Cytopathic effect was read 4–5 days after inoculation and TCID_50_ was calculated as previously described [28].

### Brain immunohistochemistry and confocal imaging

Infected mice were euthanized at 9 dpi and perfused with sterile, 1 x PBS (Gibco). One hemisphere was stained with hematoxylin–eosin (H&E) at Histoserv (Germantown, MD). The images were scored by a blinded pathologist. The other hemisphere of the brain from each mouse was collected and immediately placed in 4% paraformaldehyde for 24 h before being transferred into 30% (w/v) sucrose solution until the brains were fully infused. Brains were then embedded in TissueTek O.C.T (Sakura-Finetek, Torrance, CA) mounting media and 30μm sections cut using a Leica CM1900 cytostat (Leica Biosystems, Buffalo Grove, IL), mounted onto Superfrost Plus microscope slides (Fisher Scientific) and stored at −80^◦^C. For immunofluorescence-immunohistochemistry (IF-IHC), slides were warmed and allowed to dry at room temperature. The sections were then hydrated in 1 × PBS (Gibco), followed by antigen retrieval in sodium citrate buffer, pH 9.0 at 80°C for 8 and 10 min of cooling. The sections were then permeabilized in Triton X-100 for 1 h at room temperature (RT). TUNEL staining was performed after antigen retrieval as per manufacturers’ instructions (EMD Millipore ApopTag-FITC kit), followed by IF-IHC co-staining. For IF-IHC, sections were incubated for at least 1 h at RT in 5% normal goat serum, diluted in 1% bovine serum albumin and 0.05% Triton X-100 in 1 x PBS to block non-specific binding. The sections were then stained with combinations of anti-NeuN (EMD Millipore, Burlington, MA), anti-CD45 (BD Biosciences, San Jose, CA) and Rabbit-anti-Ebola GP (clone: KZ52; Absolute antibody, Boston, MA). Primary antibodies were diluted in buffer (1% BSA + 0.05% Triton X-100 in PBS) and applied for 24 h at RT. Sections were then incubated with species specific highly cross-absorbed Alexa-fluor conjugated (ThermoFisher, Carlsbad, CA) goat- anti-IgG secondary antibodies in dilution buffer for at least 2 h at RT. All slides were then mounted with Prolong Diamond Anti-Fade mounting media containing DAPI (ThermoFisher, Carlsbad, CA). Whole section images were acquired using an Olympus VS-120 Virtual Microscope, using Olympus VS software (Olympus LSS).

### Flow cytometry

Brain tissues were collected after intracardiac perfusion with sterile PBS, and digested in RPMI 1640 containing 0.1% Trypsin plus 0.015% DNase I for 30 min at 37 °C with pipetting every 10 min. Cells from brain were resuspended in 10 mL of 30% Percoll (GE Healthcare, Chicago, IL) and then underlaid with 1 mL of 70% Percoll. After centrifugation at 800 x g for 30 min, cells were collected from the 30%-70% interface, washed in RPMI 1640 and isolated by centrifugation at 400× g for 5 min. This method is optimized for collecting microglia and infiltrating mononuclear cells [28]. Splenocytes were obtained by dissociating the spleen into single-cell suspensions using a 70 μm cell strainer. Cells were resuspended in ACK lysis buffer to remove red blood cells. Isolated cells were stained with a cocktail of antibodies against various lineage markers, including CD45 (30-F11), F4/80 (BM8), CD11b (M1/70), CD11c (N418), Ly6G (1A8), NK1.1 (PK136), CD3 (145-2C11) and CD69 (H1.2F3). For intracellular staining, cells were fixed with 2% paraformaldehyde for 12 min on ice, permeabilized using 0.5% saponin in PBS, and stained with antibodies specific to IFNγ (XMG1.2) for 1 h on ice. Antibodies were purchased from Biolegend or BD Biosciences. Flow cytometry data were collected with the BD LSRFortessa X-20 flow cytometry (BD Biosciences), and the results were analyzed using FlowJo software (version 10).

### RNA extraction

After perfusion, the brains were collected into Trizol from infected animals at 9 dpi and stored at −80 °C until RNA was isolated (per Trizol manufacturers’ protocol). RNA concentration and purity were determined by spectrophotometry at 260 and 280 nm using a NanoDrop 1000 spectrophotometer (ThermoFisher).

### Gene expression analysis

mRNA expression levels of immune-related genes were determined using the Nanostring nCounter gene expression system (NanoString Technologies, Seattle, WA). Isolated RNA (100 ng) was hybridized with probes from nCounter Mouse Inflammation v2 Panel at 65 °C for 18 h. Hybridized products were prepared for cartridge loading on an nCounter PrepStation. Digital Counting of fluorescent signals was conducted using the nCounter Digital Analyzer. Data analysis including statistics was carried out with the nSolver 4.0 software.

### Behavioral tests

Behavioral tests were conducted as described [29]. Briefly, the Open Field Test (OFT) assessed horizontal exploratory locomotor activity and anxiety behaviour. Mice were placed in the corner of a square arena (40 × 40 cm) and allowed to explore freely for 30 min. The trial was recorded with an overhead camera; total distance travelled, and time spent in the centre of the arena (interior 50%) were automatically scored using Any-maze software (Stoelting Co., Wood Dale, Il). Anxiety-like behaviours was assessed using the Elevated Plus Maze (EPM), a raised maze (50 cm above the ground) with two enclosed arms (35 × 5 × 15 cm) perpendicular to two open arms (39.5 × 5 cm) intersected by an open central area (5 × 5 cm). Mice were placed in the centre of the maze facing one of the two open arms and recorded with an overhead camera as they freely explored the maze for 10 min. Time spent in the closed and open arms and total distance travelled was determined using Any-maze. The rotarod test was used to assess locomotor coordination and endurance. Mice were placed on rotating drum (1¼ in diameter) for 3 min with accelerating speed ranging from 5 to 20 rpm. The duration that the mouse stayed on the rotating rod was recorded. The average latency time to fall for the three trials was used for analysis. If the mouse did not fall, a time of 180 sec was recorded. In addition, Novel Object Recognition (NOR) tests were performed to evaluate cognition and memory; a full description is available in the supplementary data section.

### Statistical analysis

Comparisons between groups used the 2-tailed unpaired Student’s *t* test or mixed-effects analysis with Dunnett’s multiple comparisons as appropriate; *p*-values were adjusted for multiple comparisons as appropriate. Survival curves were tested using the Log-rank (Mantel–Cox) test. Statistical analyses were conducted with GraphPad Software (version 7.03). Adjusted *p < 0.05* was considered statistically significant.

## Results

### Lethal meningoencephalitis in neonatal C57BL/6 mice challenged with VSV-EBOV pseudovirus can be used to assess the effectiveness of therapeutics targeting the GP

**Figure 1.**
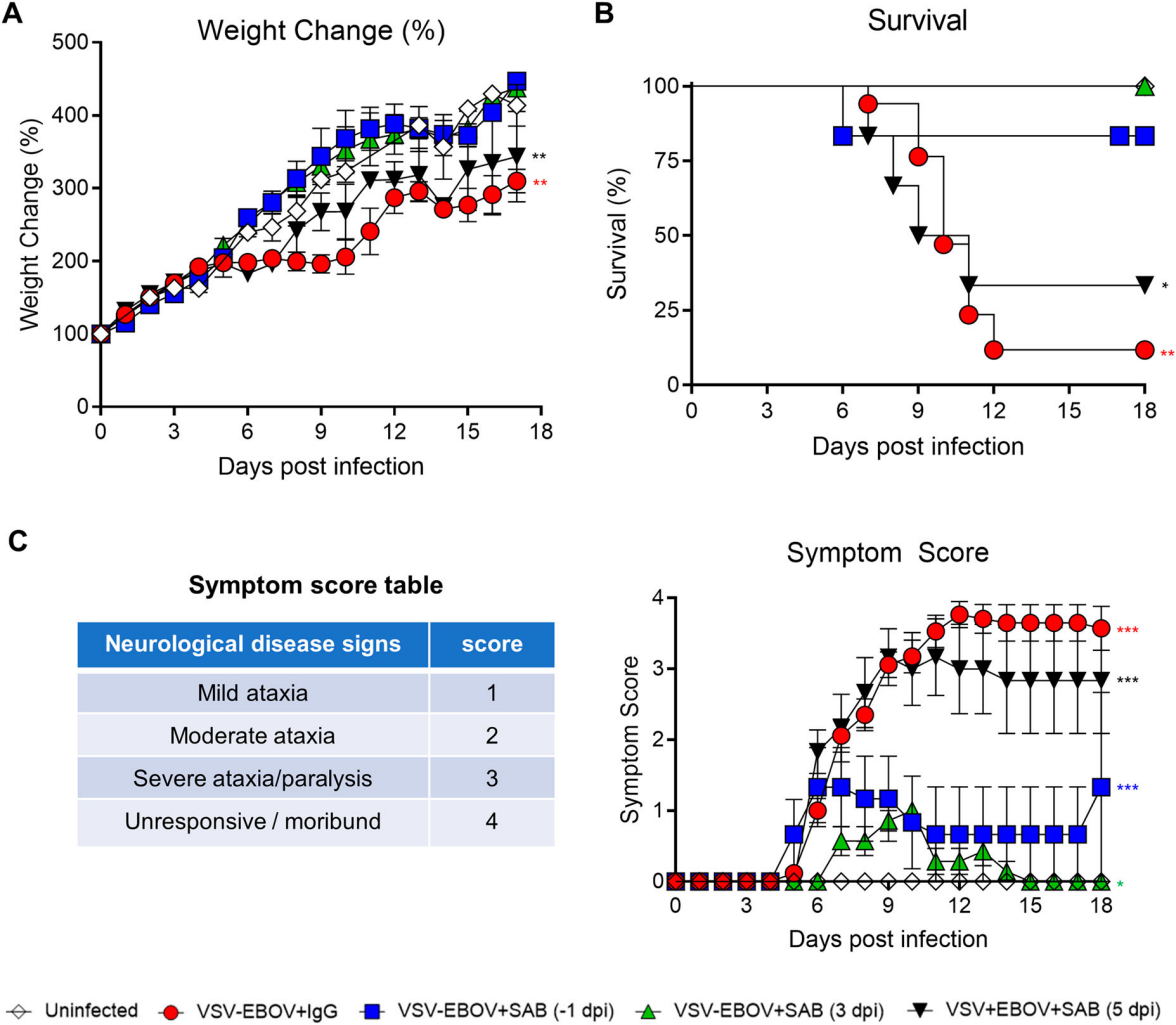
Use of VSV-EBOV model to assess therapeutics that target the EBOV-GP. P3 C57BL/6 mice were infected with 1000 TCID_50_ of VSV-EBOV and treated with human IgG isotype controls (IgG control; 100 mg/kg) or SAB-139 (100 mg/kg) prophylactically (−1 dpi), or therapeutically (3 or 5 dpi) (*n *= 6–7/group). Controls include uninfected and control Ab-treated animals (*n *= 28 and *n *= 17, respectively). Mice were monitored for weight changes (a), survival (b) and neurological symptoms (c). Symptom scores are 1 (staggering, broadened stance), 2 (moderate ataxia), 3 (severe ataxia/paralysis), and 4 (unresponsive/moribund). Data are shown as mean ± S.E.M. and represent 2–6 independent experiments. Statistical significance was determined using mixed-effects analysis with Dunnett’s multiple comparisons (a and c) and the log-rank test (b), respectively. *P < .05, **P < .01, ***P < .001 compared to uninfected mice.

Previous studies examining the administration of VSV-EBOV to neonatal mice had shown that vaccination of 3-day old C57BL/6 mice with VSV-EBOV pseudovirus results in a lethal meningoencephalitis [25]. As previously described [25], mice showed reduced weight gain, as well as severe neurological disease that manifests as tremors, widened stance, and ataxia that progresses to seizures, difficulty in righting up, paralysis, and eventually death 7–12 dpi (Figure 1(a–c)). To explore whether this BSL-2-permisible animal model could be adapted to test therapeutics targeting the GP, neonatal mice challenged on P3 with VSV-EBOV (1000 TCID_50_; s.c.) were treated with polyclonal SAB-139 (100 mg/kg; i.p.), which targets EBOV-GP, or IgG isotype controls at −1, 3, or 5 dpi. As shown in Figure 1, SAB-139 treatment given at −1 or 3 dpi normalized weight gain, lower neurological disease severity scores, and improved survival. On the other hand, SAB-139 treatment at 5 dpi marginally reduced the severity score and improved survival as compared to IgG control-treated mice (Figure 1(a–c)). These data suggest that treatment with SAB-139 up to 3 dpi reduces the neurological infection in mice that received VSV-EBOV s.c., however, the antibodies become less effective when administered in the later stage of the disease.

### SAB-139 treatment reduces virus titres and brain pathology following VSV-EBOV infection

**Figure 2.**
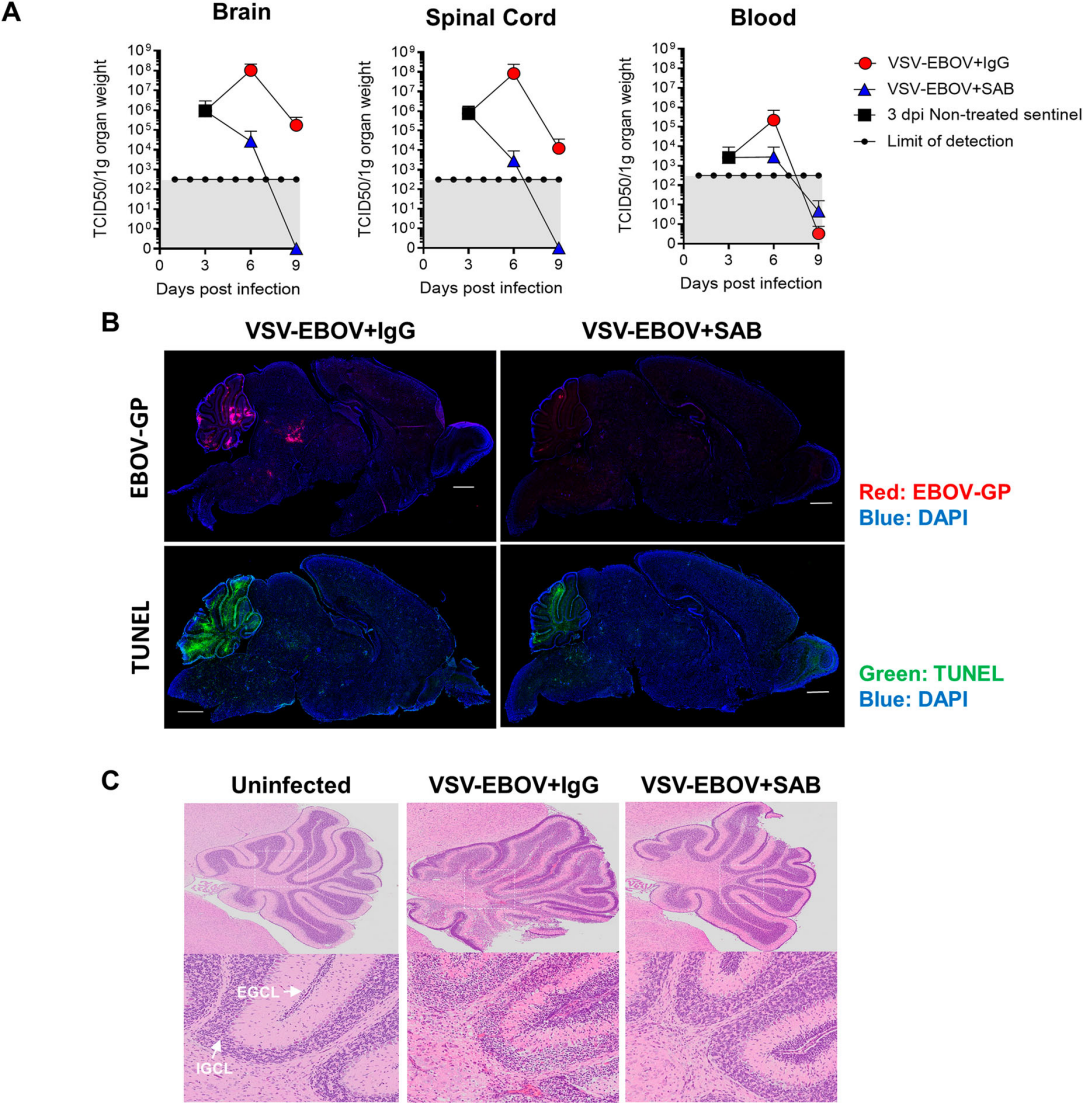
Viral burden and lesions in the brain of VSV-EBOV-infected mice. (a) VSV-EBOV-infected mice were treated with SAB-139 or IgG control (100 mg/kg) at 3 dpi. Viral loads were evaluated by TCID_50_ in the brain, spinal cord and blood of 3 (pre-treatment), 6 and 9 dpi mice treated with SAB-139 or IgG control (*n *= 4–6/group). Data are shown as mean ± S.D. and represent 2–3 independent experiments. (b) Viral antigen and apoptotic cell markers in the brains of VSV-EBOV-infected mice treated with IgG control (VSV-EBOV + IgG; *n *= 4) or SAB-139 (VSV-EBOV + SAB; *n *= 4) at 3 dpi, and sacrificed at 9 dpi. The images show representative immunofluorescence staining for EBOV-GP antigen (red), TUNEL (green) and DAPI (blue) in brain sections. Scale bar: 1 mm. (c) Histopathology of the cerebellum of uninfected mice (*n *= 2), and VSV-EBOV-infected mice treated with IgG control (VSV-EBOV + IgG; *n *= 4) or SAB-139 (VSV-EBOV + SAB; *n *= 4). The upper images show representative H&E staining of cerebellum (40×). The bottom close-up images show representative brain lesions observed in cerebellum. EGCL, the external granular cell layer; IGCL, the internal granular cell layer.

EBOV is known to be neurotropic, and limited data suggests that it can persist in the CNS for extended periods [30]. Thus, understanding whether antivirals reduce the viral load in these critical tissues is important in selecting therapeutic candidates. Consistent with the previous study [25], replicating VSV-EBOV was evident in blood, as well as spinal cord and brain as early as 3 dpi and peaked by 6 dpi (Figure 2(a)). Viral antigen and apoptosis were primarily evident in pons, midbrain and cerebellum of brains of infected mice that received IgG controls at 9 dpi. Examination of the infected cerebella showed broadening of the subarachnoid space with hyperplasia of the external granular cell layer (EGCL), reduced cellularity of the internal granular cell layer (IGCL), and perivascular cuffing of mononuclear cells in IgG control-treated mice (Figure 2(c) and Table 1). In addition, foci of picnotic nuclei in the cerebellum confirm the increased levels of apoptosis (Figure 2(b,c)). Along with the improved survival, SAB-139 treatment at 3 dpi significantly reduced the viral load in the brain, spinal cord and blood from infected mice (Figure 2(a)), however, accumulation of viral antigen particularly in midbrain and pons as well as increased TUNEL^+^ cells were still evident in the brains of SAB-139-treated mice albeit at lower levels than in IgG control-treated mice (Figure 2(b)). Similarly, at 9 dpi the cerebella of SAB-139-treated mice showed milder EGCL thickening, IGCL thinning, and perivascular cuffing (Figure 2(c) and Table 1).

**Table 1.** Summarized pathology of the brain.

	EGCL thickness	IGCL cellularity	Perivascular cuffing	Picnotic nuclei
VSV-EBOV + IgG	6–10	Reduced	Midbrain, Cerebellum, Thalamus, Colliculus, Pons, Brain stem	EGCL and IGCL
VSV-EBOV + SAB	1–7	n/a	Midbrain, Brain stem, Cortex, Thalamus, Corpus callosum	n/a
Uninfected	1–3	n/a	n/a	n/a

### SAB-139 treatment attenuates VSV-EBOV-induced microglial loss and cell infiltration into the brain

**Figure 3.**
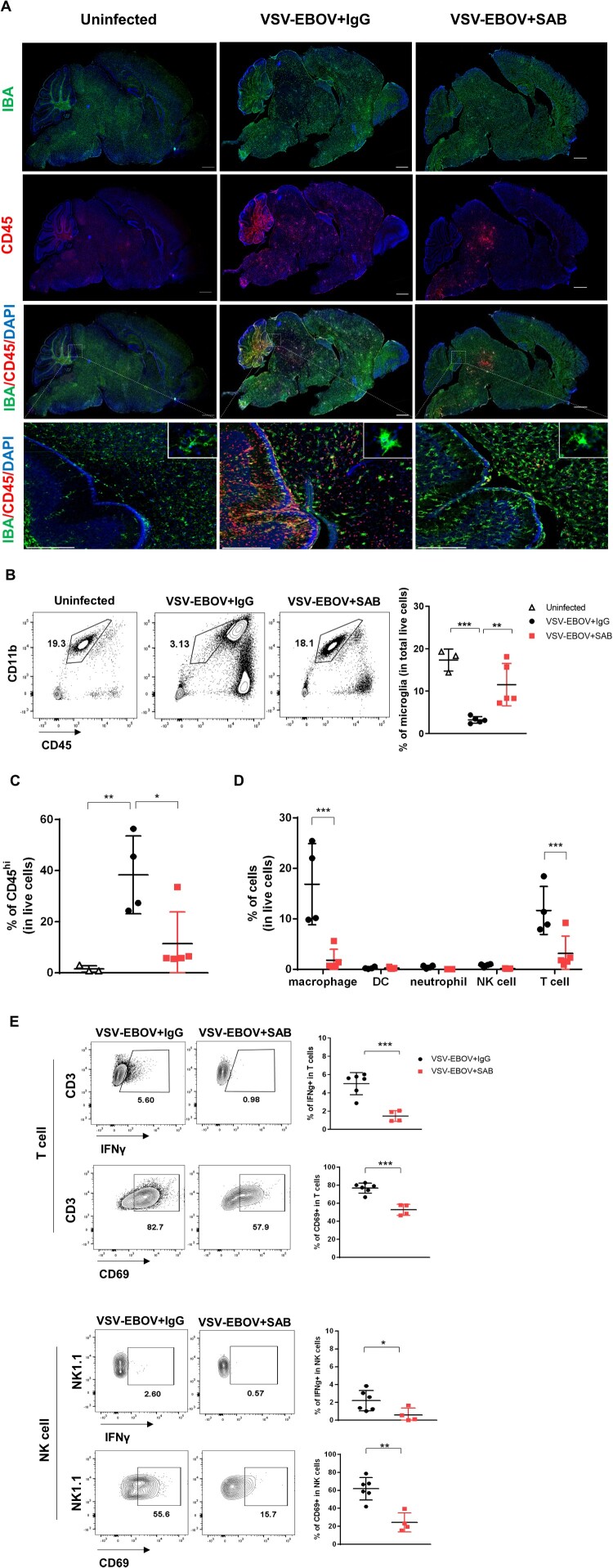
Microglial loss and immune cell infiltration in the brains of VSV-EBOV-infected mice. (a) VSV-EBOV-infected mice were treated with IgG control (VSV-EBOV + IgG; *n *= 4) or SAB-139 (VSV-EBOV + SAB; *n *= 4) at 3 dpi, and then sacrificed at 9 dpi. The images show representative immunofluorescence staining for IBA (green), CD45 (red) and DAPI (blue) in brain sections. Inset: Close-up images of microglia. Scale bar: 1 mm (in whole brain images) or 25 μm (in the bottom images). (b–d) Flow cytometry analysis of cells isolated from the brain at 9 dpi. Representative plots showing the population of microglia in the brain (b, left; 1 of 3-5 mice with similar results). The graphs show the percentage of microglia (CD45^int^CD11b^+^) (b, right) and CD45^hi^ infiltrating cells (c) in total live cells, respectively. The percentage of macrophages (CD45^hi^CD11b^+^F4/80^+^), DCs (CD45^hi^CD11c^+^F4/80^-^Ly6G^-^), neutrophils (CD45^hi^CD11b^+^Ly6G^+^F4/80^-^), T cells (CD45^hi^CD3^+^NK1.1^-^) and NK cells (CD45^hi^ NK1.1^+^CD3^+^) in total live cells at 9 dpi (d). (e) IFNγ and CD69 expression in T cells (CD45^hi^CD3^+^NK1.1^-^) and NK cells (CD45^hi^NK1.1^+^CD3^-^) from the brains at 9 dpi. The cells were incubated with brefeldin A (10 μg/mL) for 3.5 h to measure IFNγ. Data are representative of two independent experiments. Data shown as means ± S.D. (*n*=3-6). *P<0.05, **<0.01.

Results from the PREVAIL trial that is following survivors of EBOV infections for 5 years show that many survivors have neurological sequelae and virus has been recovered from the CNS months after infection [7]. Although our understanding of the effects of the virus on the CNS is limited, previous studies in macaques show that EBOV causes local inflammation, neuronal apoptosis, and microglial activation in the dorsal ganglia [31]. In our model at the peak of disease (9 dpi), the brains of mice challenged with VSV-EBOV showed a significant increase in CD45^hi^ infiltrating cells including macrophages (CD45^hi^ CD11b^hi^), DC, T cells, neutrophils and natural killer (NK) cells, as well as a marked reduction in CD45^int^CD11b^hi^ microglial cells (Figure 3(a–d)). The infected brains showed multiple foci of CD45^hi^ cells throughout the brain, even in areas that are not heavily infected, but the heavily infected tissues such as cerebellum, midbrain and pons showed a marked depletion of IBA^+^ microglial (Figure 3(a,b)). While SAB-139 treatment lessened the reduction in IBA^+^ cells in the brains, it failed to prevent microglial activation as evident by increased IBA staining and amoeboid morphology that is observed in infected mice (Figure 3(a)). Treatment with SAB-139 also reduced cellular infiltration, with foci of CD45^+^ cells limited to the mid and hind brain (Figure 3(a,c)) as well as a fewer infiltrating macrophages and T cells relative to IgG control-treated mice (Figure 3(d)), suggesting an overall reduction in inflammation of the brain. Further, T cells and NK cells infiltrating the brains of SAB-139-treated animals displayed a less active phenotype with a smaller fraction producing IFNγ or expressing CD69 (Figure 3(e)). Together, this suggests that SAB-139 treatment can modulate the disease course even when administered after the virus is established in the brain, resulting in reduced cellular infiltration, fewer activated lymphocytes and reduced microglial loss.

### SAB-139 treatment reduces overall inflammatory profile in the brain

**Figure 4.**
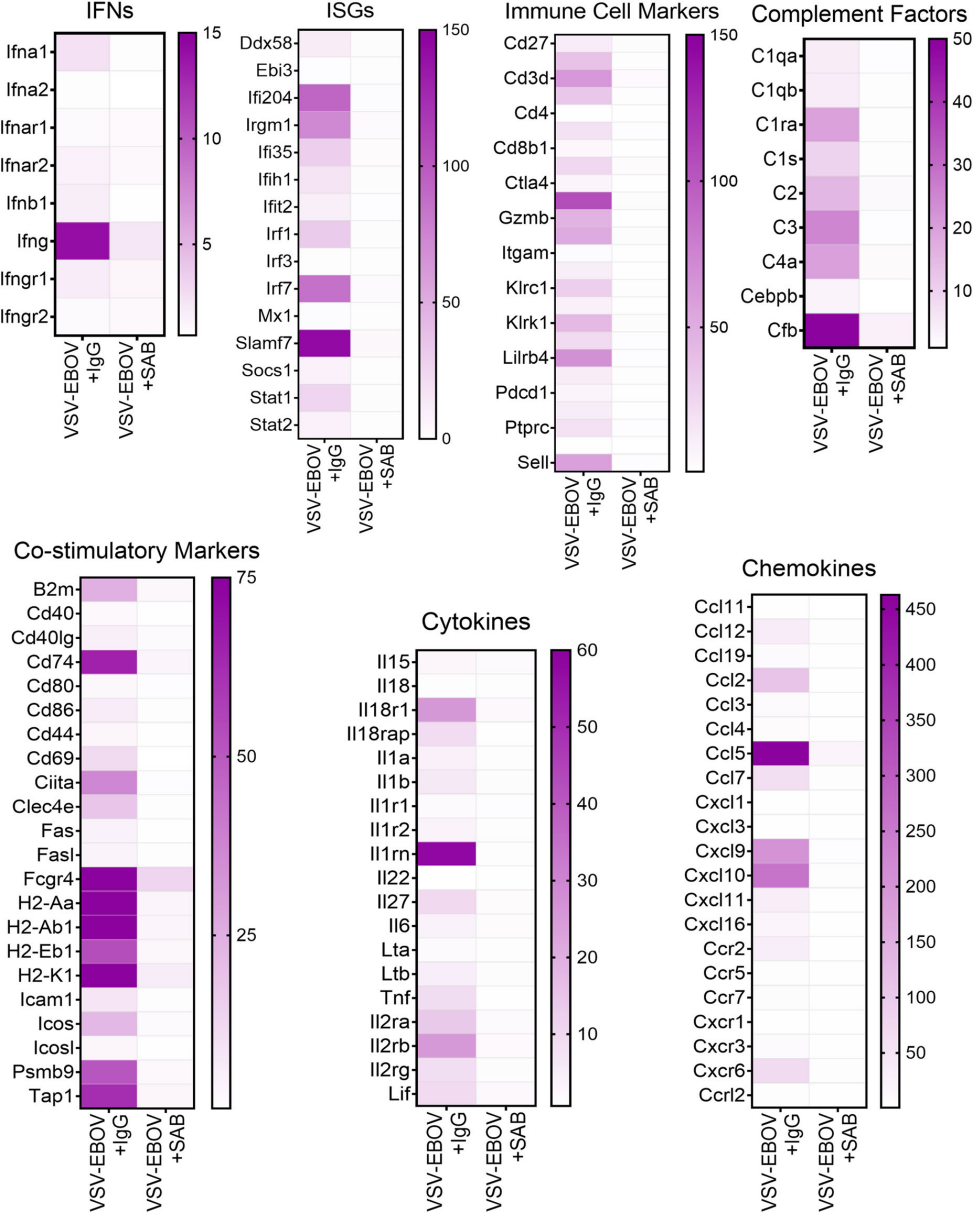
Gene expression in the brains of VSV-EBOV-infected mice treated with IgG control or SAB-139. Heat map of mRNA expression in the brain of VSV-EBOV-infected mice treated with IgG control (VSV-EBOV + IgG; *n *= 4) or SAB-139 (VSV-EBOV + SAB; *n *= 4) at 9 dpi. RNA was assessed by NanoString. Genes selected based on significant changes by SAB-139 treatment from a panel of 596 genes and organized by gene function. A total of 596 gene expression is available in Figure S4.

Uncontrolled inflammation is thought to be characteristic of EVD. Assessment of mRNA expression in the brains of the infected mice showed that the expression of genes associated with immune cell activation is highly upregulated (Figure 4). This includes genes associated with interferon responses (e.g. *Ifna1, Ifng, Ifi204, Irgm1, Irfs and Slamf7*), activation of antigen presenting cells (e.g. *Mhc*, *Ccl2*, *Cd74*, *Ccl5*, *Cxcl10* and *Cxcr6*), and recruitment and activation of T and NK cells (e.g. *granzymes*, *Il2r*, *Il-18r1*, *Il27*, *Ccl5*), as well as genes linked to complement activation (e.g. *C1qa, C1qb, C1ra, C1s, C2, C3, C4a, Cebpb, and Cfb* (Figure 4). Consistent with the reduced viral load and restricted inflammation, we observed that SAB-139-treated mice have reduction in the inflammatory response in the brain parenchyma (Figure 4). Further, GAGE analysis showed that VSV-EBOV infection of the brain leads to the upregulation of immune effector process, defense response, antigen processing and presentation whereas it causes the downregulation of cell development regulation, cell fate commitment, erythrocyte homeostasis and neurogenesis regulation (Figure S1 and Table S1). Treatment with SAB-139 in turn, reduced changes in gene expression linked to the immune response, cell fate, tissue remodelling, and neurogenesis (Figure S1 and Table S1). Together, these findings suggest that SAB-139 treatment effectively reduces the infection and lessens the immune and inflammatory responses, thereby limiting immune-related damage to the brain.

### SAB-139 treatment prevents VSV-EBOV-induced long-term motor and behaviour defects

**Figure 5.**
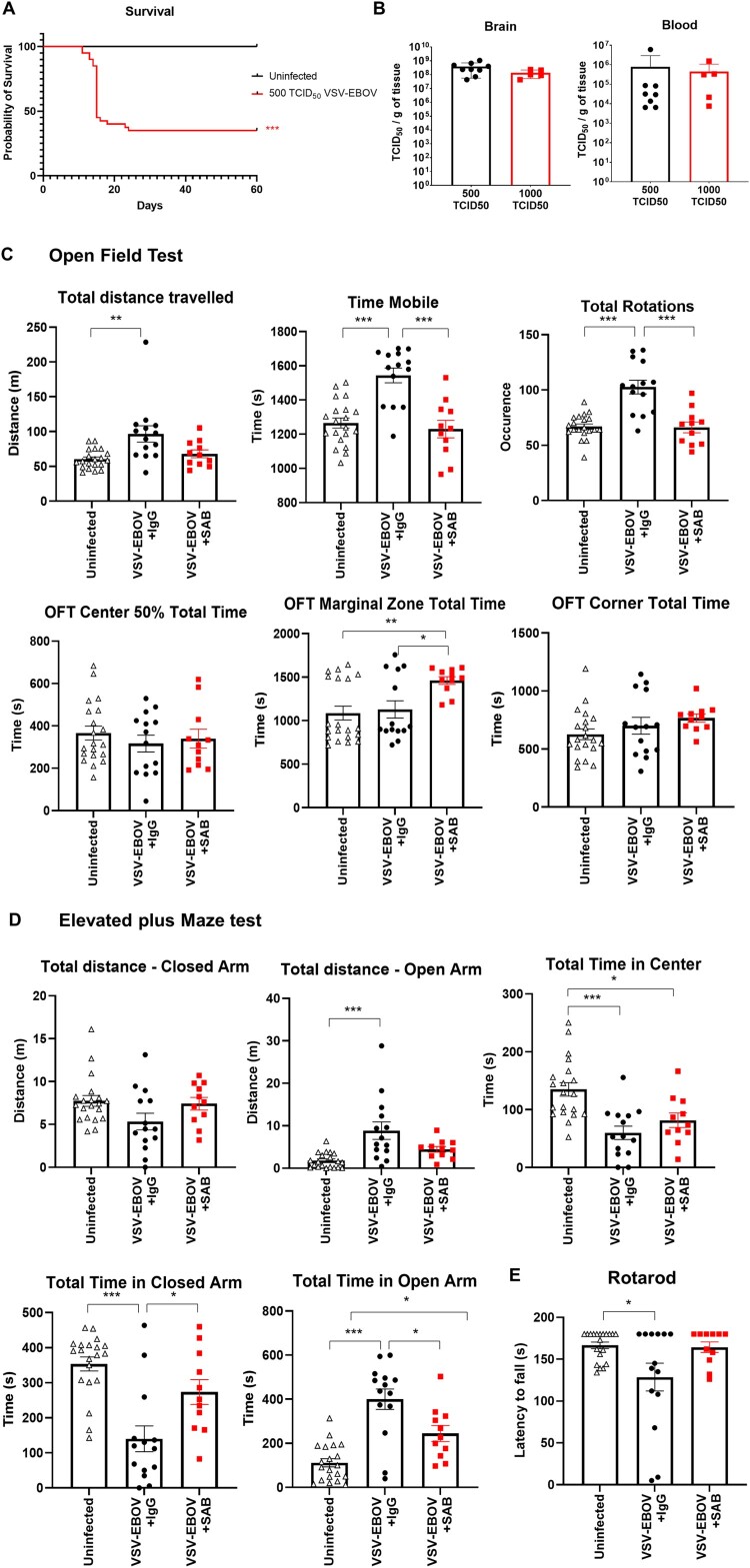
Use of low-challenge VSV-EBOV model to assess the impact of treatment on long-term neurological sequelae. P3 mice were s.c. infected with 500 TCID_50_ of VSV-EBOV and monitored for survival (a) for 60 days. Statistical significance was determined using the log-rank test. ***P<.001 compared to uninfected mice. (b) Comparison of viral load in the brain and peripheral blood from mice challenged with 500 TCID_50_ or 1000 TCID_50_ of VSV-EBOV at 6 dpi by TCID_50_ analysis. Data are shown as mean ± S.D. and represent 2–3 independent experiments. (c) Behavior tests in uninfected (*n *= 20), VSV-EBOV-infected mice (VSV-EBOV; *n *= 14) or VSV-EBOV-infected mice treated with SAB-139 (VSV-EBOV + SAB; *n *= 11) at six months post-infection. Open Field Test (OFT) shows total distance travelled, total time spent moving and occurrence of mouse rotation as well as time within the center 50%, marginal and corner area of the OFT maze. (d) Elevated Plus Maze (EPM) shows total distance travelled in the closed and open arm as well as time spent in the center, closed and open arm of the maze. (e) Rotarod test shows average latency to fall (mean of 3 test/mouse) balancing on a rotating rod of gradually increasing speed over 180 s. *P < .05,  *P < .01.

Several reports indicate that EVD survivors suffer from neurologic complications such as headaches, memory loss, tremor, seizures and strokes over the long term [32]. To date, it is not known whether these long-term sequelae are reduced in EVD survivors treated with the recently licensed monoclonal Abs to EBOV-GP. In our model, infected mice treated with SAB-139 had reduced tissue damage, but still showed evidence of neuroinflammation. To develop an animal model to assess the long-term consequences of GP-mediated viral neurotropism, we titered down the challenge and determined that inoculating neonatal mice with 500 TCID_50_ VSV-EBOV consistently allows for 30-40% survival despite attaining similar viral burden in the brain and blood at the peak of infection as with the mice that received 1000 TCID_50_ VSV-EBOV (Figure 5(a,b)). We reasoned that this sublethal challenge dose would allow us to assess the long-term effects of the infection and explore the effect of therapeutic candidates on sequela. To examine sequela from their early infection using a sublethal-challenge, convalescent 6 months-old mice treated with SAB-139 or a control IgG antibody at 3 dpi were subjected to the OFT, EPM and rotarod tests to evaluate their locomotor activity and behavioural changes. As shown in Figure 5(c), VSV-EBOV-infected mice displayed increased distance travelled, motility and rotation time in the OFT compared to age-matched uninfected mice, suggesting a hyperactive phenotype in VSV-EBOV-infected mice. In the EPM test, these mice spent more time and travelled a greater distance in the open arm than uninfected controls, suggesting that hyperactivity was paired with reduced anxiety and thigmotactic tendencies (Figure 5(d)). Lastly, the rotarod test showed a reduction in latency to fall in infected mice compared to age-matched controls, indicating an impairment in motor function and coordination (Figure 5(e)). Of note, the NOR test used to assess cognition and memory did not show significative changes due to infection (Figure S2). Importantly, none of these traits were evident in the mice that were treated with SAB-139 at the onset of infection (3 dpi) (Figure 5(c–e)) indicating that controlling the virus by targeting the glycoprotein is sufficient to reduce neurological sequelae, although the mice spent significantly more time in the marginal zone in the OFT and in the open arm of the EPM. Together, these data suggest that a low-challenge model of infection driven by EBOV-GP can be used to assess a long-term sequela and model the impact of therapeutics targeting EBOV-GP.

## Discussion

Several reports have established that EBOV is neurotropic and survivors of EVD can harbour live virus in immune privileged sites like eyes long after recovery [5,33,34]. The potential for neurotropism of EBOV in neonatal mice was established early on in studies by Pattyn et al. and Van der Groen et al. which showed that EBOV caused a lethal meningoencephalitis [35,36]. In this study, we describe a BSL-2 compatible animal model that can be used to explore the dynamics of EBOV-GP in the CNS and test potential therapeutics that target it. Our results show that P3 C57BL/6 mice challenged with a replication-competent VSV-EBOV pseudovirus develop a lethal meningoencephalitis characterized by weight loss and ataxia. Replicating virus is evident in peripheral blood, spinal cord, and the brain within 3 days of challenge and peaks at 6 dpi. Within the brain, VSV-EBOV primarily localizes to mid and hind brain where it causes an increase in local cell death, inflammation, and cellular infiltration. By 9 dpi, when the mice die, there is evidence of edema of the subarachnoid space, thickening of the external granular layer, and cell loss in the inner granular cell layer of cerebellum, as well as increased apoptosis and microglial loss in midbrain and pons. The infected brains show scattered foci of cellular infiltration with increased expression of interferon-inducible genes. Mice that receive a lower viral challenge develop neurological symptoms but have reduced lethality. These animals show hyperactivity, reduced anxiety and impairment in motor coordination and endurance as late as 6 months after challenge. We used a polyclonal antibody, previously shown to target EBOV-GP, to determine if the model can be used to screen therapeutics. We show that treatment as late as 3 dpi reduces the levels of virus in the brain, spinal cord and blood, and lowers the inflammation, as evidenced by reduced number of infiltrating immune cells and fewer activated lymphocytes expressing lower levels of IFNγ and CD69. Reduced cellular infiltration and inflammation in turn, is associated with a reduction in microglial loss and apoptosis compared to IgG control-treated infected mice. Importantly, SAB-139-treated animals survive the challenge and have reduced neurological sequelae.

Although the clinical presentation and evolution of the EVD is well described, due to the sporadic nature of the outbreaks and the high infectivity and lethality of the virus, there is limited clinical data on host–pathogen interactions, in depth characterization of the immune response to EVD, or established surrogates of protection. The paucity in animal models and requirement that all studies be performed in high containment labs has further hindered investigations into pathogenesis of EBOV and its treatment. Currently, NHP are considered the model of choice as it recapitulates some aspects of the human disease, but studies are difficult, costly and time consuming, and raise important ethical considerations. As a result, most studies involve only a few specimens and selected measurements. Wild-type EBOV does not cause pathology in immune competent mice, so most studies in rodents have used either severely immunodeficient animals or mouse adapted virus. Neither model replicates the haemorrhage seen in EVD, but the MA-EBOV infection models present with cytokine release, organ damage and death [37]. Additionally, newer models using humanized mice, ferrets and guinea pigs require a BSL-4 environment. The development of a symptomatic model in mice, that can be used in BSL-2 to examine EBOV-GP-driven tropism and the therapeutics that target it, offers some advantages, allowing for easier screening of therapeutics, alone or in combination, and initial pharmacokinetic/pharmacodynamic studies. Further, the availability of a symptomatic sublethal model may facilitate research into the establishments of viral reservoirs in the CNS and long-term neurological sequelae of EBOV, a problem that has become evident as new therapies for EBOV increase the number of survivors. Moreover, a model that is not immediately lethal, may eventually serve as a platform to map potential mutations resulting from selective pressure of specific antibodies to EBOV-GP *in vivo*. Of note, as most models of haemorrhagic fever, the infection appears to primarily involve the CNS, and although there is increased mRNA expression of IL-1, 6 and 8 and TNFα, the pattern of gene expression does not suggest a cytokine storm. Lastly, the extensive immune cell infiltration together with the ingress of therapeutic Abs (Figure S3) suggests that the VSV-EBOV causes a disruption of the endothelial barriers and further studies will need to establish whether this mimics the clinical impact of EBOV infection.

Several studies have shown that the VSV-EBOV vaccine is not neurotropic in adult mice [25,38]. The mechanisms underlying the increased susceptibility to neurotropism in young mice are unknown but may be rooted in the high frequency of mitotically active immature neurons and neuronal precursors, active pruning, and differential expression pattern of cellular receptors, which may be used by the virus to gain entry [39,40]. Alternatively, mildly impaired immune responses to the virus in the first days of life can result in unchecked virus proliferation [41], however as previously shown, administration of Poly I:C can increase interferon levels leading to control of the challenge, indicating that P3 mice can mount effective IFN responses [25]. Together these data suggest that the increased susceptibility of neonatal mice to neurotropic infection with VSV-EBOV may be developmental, rather than linked to defects in the neonatal immune system. Lastly, it is interesting to speculate that mice received a lower challenge had increased survival despite eventually reaching a similar peak viral load in CNS by 6 dpi, and mice treated after 5 dpi could not be rescued by the SAB-139 antibodies, suggesting that early tissue damage may be linked to lethality in this model.

EVD survivors report headache, fever, memory loss, hearing loss, tremor, seizure and neurocognitive disorders, that can persist for more than 1 year after acute EVD or discharge from Ebola treatment centre [8,32,42,43]. In agreement with this, neonatal mice infected with VSV-EBOV exhibit persistent neurological sequelae such as hyperactivity and impaired coordination that remain evident more than 6 months after infection. The behavioural sequela observed are consistent with the infection, inflammation (including increased expression of complement components [44]), and neurodegeneration in midbrain and cerebellum that are accompanied by activation and loss of microglia in acutely infected neonatal mice that could contribute to the long-term sequelae as midbrain microglia are known to play a role in protecting dopaminergic neurons from inflammation-induced cell death by triggering immune-suppressive responses [45]. Of note, the loss of dopaminergic neurons in the midbrain is a pathological hallmark of Parkinson’s disease (PD) [46], and PD symptoms such as rigidity, retropulsion and shuffling gait have been reported in EVD survivors [32]. Future studies will need to explore whether VSV-EBOV-induced microglial loss contributes to neurodegeneration and subsequent neurological complications, or it is secondary to neurodegeneration and inflammation, as this may help identify therapeutic targets for these patients beyond antivirals. Interestingly, there is evidence of extensive microgliosis in the same area of the mice treated with SAB-139 as well as a residual increase in complement-related factors. Future studies will need to address whether the microgliosis is transient and whether it can mediate presynaptic membrane damage of the hippocampus resulting in long-term memory loss and cognitive dysfunction as described in patient with encephalitis due to West Nile virus [47].

Consistent with previous findings showing that SAB-139 controls the infection in mice challenged with MA-EBOV as well as NHP challenged with EBOV [26,27], we found that administration of SAB-139 prevents weight loss and severe neurological symptoms leading to improved survival in VSV-EBOV-infected mice. Interestingly, the antibodies improved the outcome even when administered after the infection had reached the CNS (3 dpi), as SAB-139 treatment significantly lowered viral titres, and reduced local inflammation, microglia loss, and immune cell infiltration in the brain of infected mice. Importantly, this reduced long-term neurological deficits in surviving mice. Treatment of infectious diseases that affect the CNS is often limited by the accessibility of therapeutics to the CNS, which in turn is dependent on the permeability of the blood brain barrier (BBB). In our study, the intraperitoneally injected Abs were detected in the brain tissue in the infected, but not in the uninfected ones (Figure S3). Along with this, the reduction in viral loads in the brain at a time when there is little virus found systemically make it tempting to speculate that at least part of the therapeutic effect of the antibody could be local, however, the mechanisms involved may be more complex. If penetrating the CNS is critical for its activity, future studies will need to examine whether its therapeutic effect is replicated in the CNS of EBOV-infected humans. Lastly, it is well established that the anti-microbial effect of antibodies is primarily mediated through the binding of FcγR on myeloid and NK cells, and although the discordance between species is modest and mice have been frequently used to study the effector function of human IgGs, potential differences in Fc function should be considered when using mice to model human diseases.

In summary, while this BSL-2 compatible infection model cannot be considered a model of EVD, our data show that it can be used to screen candidate therapeutics targeting the EBOV-GP alone or in combination and examine the contribution of different therapeutics attributes to viral clearance in different tissues, as well as inform on GP-mediated tropism in the CNS facilitating the development of better therapeutics.

## Supplementary Material

Supplemental Material
