# Supplementary material for: Characterization of the therapeutic effect of antibodies targeting the Ebola glycoprotein using a novel BSL2-compliant rVSVΔG-EBOV-GP infection model
Source: Emerg Microbes Infect. 2021 Nov 10;10(1):2076–89. doi: 10.1080/22221751.2021.1997075 (PMC8583756; doi:10.1080/22221751.2021.1997075)
Supplement: Supplemental Material [file TEMI_A_1997075_SM3280.zip › TEMI 1997075_Supplmentary Files/Table S1.docx]

| **Pathway** | **Genes** |
| --- | --- |
| **Antigen processing and presentation** | B2m, Ccr7, Cd1d1, Cd74, Fcer1g, Fcgr1, Fcgr2b, Fcgr3, Fcgrt, H2-Aa, H2-Ab1, H2-DMa, H2-DMb2, H2-Eb1, H2-K1, H2-Ob, H2-Q10, Hfe, Icam1, Ifng, Mr1, Nod2, Psmb9, Relb, Tap1, Tapbp, Traf6 |
| **Cell fate commitment** | Batf, Bcl2, Casp3, Ctnnb1, Eomes, Gata3, Gfi1, Il23a, Il4, Il7, Irf4, Itgb1, Notch1, Notch2, Olig1, Olig2, Pax2, Pml, Pparg, Prdm1, Rag2, Smad5, Stat6, Tal1, Tgfb2, Tgfbr1, Trp53 |
| **Cell junction organization** | Bcl2, Cd9, Csf1r, Ctnnb1, Fn1, Itga5, Itgb1, Smad3, Src, Tgfb1, Tgfb2, Tgfb3, Thy1, Vegfa |
| **Cell surface** | Abcb1a, Adgre1, App, Aqp4, B2m, Bst2, Btla, C1qbp, Ccl19, Ccr4, Ccr5, Ccr7, Cd14, Cd19, Cd1d1, Cd2, Cd22, Cd226, Cd244a, Cd24a, Cd27, Cd274, Cd28, Cd34, Cd36, Cd3e, Cd4, Cd40, Cd40lg, Cd44, Cd46, Cd48, Cd5, Cd53, Cd55, Cd59b, Cd6, Cd69, Cd74, Cd79a, Cd79b, Cd80, Cd83, Cd86, Cd8a, Cd8b1, Cd9, Clec5a, Cr2, Csf1r, Ctla4, Ctss, Cx3cl1, Cxcl10, Cxcl12, Cxcl9, Cxcr2, Cxcr3, Cxcr4, Dpp4, Fas, Fasl, Fcer1a, Fcer1g, Fcgr1, Fcgr2b, Fcgr4, H2-Aa, H2-Ab1, H2-Eb1, H2-K1, H60a, Icam1, Icos, Icosl, Ifng, Igf2r, Il12rb1, Il12rb2, Il13, Il15, Il17a, Il17rb, Il1a, Il1r1, Il1rl1, Il2ra, Il2rb, Il2rg, Il4, Il6, Il6ra, Il6st, Il7r, Irak2, Itga2b, Itga4, Itga5, Itga6, Itgal, Itgam, Itgax, Itgb1, Itgb2, Kit, Klra1, Klra4, Klra7, Klrc1, Klrc2, Klrc3, Klrd1, Klrk1, Mif, Mrc1, Ms4a1, Muc1, Ncam1, Nod2, Notch1, Notch2, Nt5e, Pdcd1, Pdgfb, Pecam1, Plau, Plaur, Ptprc, Sell, Slamf1, Spn, Tfrc, Tgfb1, Tgfb2, Tgfb3, Tgfbr2, Thy1, Tigit, Tlr2, Tlr3, Tlr4, Tnf, Tnfrsf11a, Tnfrsf13b, Tnfrsf13c, Tnfrsf14, Tnfrsf1a, Tnfrsf4, Tnfrsf9, Tnfsf18, Vcam1, Vegfa |
| **Defense response** | Aqp4, B2m, Batf, Bcl2, Bcl3, Bcl6, Bst2, Btk, C1qa, C1qb, C1qbp, C1ra, C1s1, C2, C3, C4bp, C6, C8a, C8b, C8g, C9, Camp, Card9, Ccl11, Ccl12, Ccl2, Ccl20, Ccl22, Ccl24, Ccl25, Ccl3, Ccl4, Ccl5, Ccl7, Ccl8, Ccr2, Ccr4, Ccr5, Ccr7, Ccrl2, Cd14, Cd160, Cd163, Cd1d1, Cd226, Cd24a, Cd28, Cd36, Cd4, Cd40, Cd40lg, Cd44, Cd46, Cd55, Cd59b, Cd74, Cd86, Cd8a, Cfb, Cfd, Cfh, Cfi, Cfp, Chil3, Ciita, Clec5a, Cr2, Csf1, Csf1r, Ctsg, Cxcl1, Cxcl10, Cxcl11, Cxcl13, Cxcl15, Cxcl16, Cxcl3, Cxcl9, Cxcr2, Cybb, Ddx58, Defb1, Defb14, Fadd, Fcer1a, Fcer1g, Fcgr1, Fcgr2b, Fcgr3, Fn1, Foxp3, Gapdh, Gata3, H2-Aa, H2-Ab1, H2-Eb1, H2-K1, H60a, Hamp, Hc, Hif1a, Ifi204, Ifih1, Ifit2, Ifitm1, Ifna1, Ifna2, Ifnar1, Ifnb1, Ifng, Ifnl2, Il10, Il12a, Il12b, Il12rb1, Il15, Il17a, Il17b, Il17f, Il17ra, Il18, Il18r1, Il18rap, Il1a, Il1b, Il1r1, Il1rap, Il1rl1, Il1rl2, Il1rn, Il2, Il21, Il23a, Il23r, Il25, Il27, Il27ra, Il2ra, Il33, Il4, Il4ra, Il6, Irak1, Irak3, Irak4, Irf1, Irf3, Irf4, Irf5, Irf7, Irf8, Irgm1, Itgax, Itgb2, Jak2, Jak3, Kit, Klrk1, Lta, Ly86, Ly96, Map4k2, Mapkapk2, Marco, Masp1, Masp2, Mbl2, Mif, Mr1, Mx1, Myd88, Naip5, Nfkbia, Nfkbiz, Nod2, Nos2, Notch1, Notch2, Nt5e, Pml, Pparg, Prf1, Prkcd, Ptafr, Ptger4, Ptgs2, Ptpn6, Ptprc, Rela, S100a8, S100a9, Sele, Serping1, Sh2d1a, Sigirr, Smad3, Spn, Stat1, Stat3, Stat5a, Stat5b, Sting1, Syk, Tap1, Tapbp, Tbk1, Tgfb1, Tirap, Tlr1, Tlr2, Tlr3, Tlr4, Tlr5, Tlr8, Tlr9, Tnf, Tnfaip3, Tnfrsf11a, Tnfrsf14, Tnfrsf1a, Tnfrsf1b, Tnfrsf4, Tnfsf11, Tnfsf8, Tollip, Traf3, Xcl1 |
| **Developmental maturation** | App, Bcl2, Btk, Cdh5, Ctnnb1, G6pdx, Gata3, Hif1a, Il15, Il21, Notch1, Pax2, Pou2f2, Pparg, Ptgs2, Runx3, Tal1, Tgfb2, Vegfa, Xbp1 |
| **Erythrocyte differentiation** | Bcl6, Ets1, G6pdx, Gata3, Hif1a, Jak2, Kit, Mapk14, Smad5, Stat5a, Stat5b, Tal1, Vegfa |
| **Erythrocyte homeostasis** | Bcl6, Cd24a, Ets1, G6pdx, Gata3, Hif1a, Jak2, Kit, Mapk14, Smad5, Stat5a, Stat5b, Tal1, Vegfa |
| **External side of plasma membrane** | Adgre1, Aqp4, B2m, Btla, Ccl19, Ccr4, Ccr5, Ccr7, Cd19, Cd1d1, Cd2, Cd22, Cd244a, Cd24a, Cd27, Cd274, Cd28, Cd34, Cd36, Cd3e, Cd4, Cd40, Cd40lg, Cd44, Cd48, Cd5, Cd55, Cd59b, Cd69, Cd74, Cd79a, Cd79b, Cd80, Cd83, Cd86, Cd8a, Cd8b1, Cd9, Cr2, Ctla4, Cxcl10, Cxcl12, Cxcl9, Cxcr3, Cxcr4, Fas, Fasl, Fcer1a, Fcer1g, Fcgr1, Fcgr2b, Fcgr4, H2-Aa, H2-Ab1, H2-Eb1, H2-K1, H60a, Icam1, Icos, Icosl, Ifng, Il12rb1, Il12rb2, Il13, Il17a, Il1rl1, Il2ra, Il2rb, Il2rg, Il4, Il6, Il6st, Il7r, Itga2b, Itga4, Itga5, Itga6, Itgal, Itgam, Itgax, Itgb1, Kit, Klra1, Klra4, Klra7, Klrc1, Klrc2, Klrc3, Klrd1, Klrk1, Ms4a1, Ncam1, Pdcd1, Pecam1, Ptprc, Sell, Slamf1, Spn, Tfrc, Tgfbr2, Thy1, Tlr2, Tlr4, Tnf, Tnfrsf11a, Tnfrsf13b, Tnfrsf13c, Tnfrsf14, Tnfrsf4, Tnfrsf9, Vcam1 |
| **Gland development** | Ahr, Bax, Bcl2, Ccl11, Cd44, Cebpb, Chuk, Csf1, Ctnnb1, Gata3, Hif1a, Ikzf1, Il6, Jak2, Lef1, Mapk1, Notch1, Pdgfb, Pdgfrb, Plaur, Pml, Smad3, Src, Stat5a, Stat5b, Stat6, Tgfb1, Tgfb3, Tgfbr1, Tgfbr2, Tnf, Tnfrsf11a, Tnfsf11, Vegfa, Xbp1 |
| **Immune effector process** | Aicda, B2m, Batf, Bcl2, Bcl3, Bcl6, Bst2, Btk, C1qa, C1qb, C1qbp, C1ra, C1s1, C2, C3, C4a, C4bp, C6, C8a, C8b, C8g, C9, Card9, Ccl2, Ccl3, Ccl5, Ccr2, Ccr7, Cd1d1, Cd226, Cd24a, Cd28, Cd36, Cd40, Cd40lg, Cd46, Cd55, Cd59b, Cd74, Cd86, Cd8a, Cfb, Cfd, Cfh, Cfi, Cfp, Cr2, Ctsc, Ctsg, Cx3cr1, Cxcl10, Cxcl9, Ddx58, Dpp4, Eomes, Fadd, Fas, Fcer1a, Fcer1g, Fcgr1, Fcgr2b, Fcgr3, Foxp3, Gata3, Gpr183, Gzmb, H2-Ab1, H2-DMa, H2-Eb1, H2-K1, H60a, Hc, Hlx, Icam1, Icosl, Ifi204, Ifih1, Ifit2, Ifitm1, Ifna1, Ifna2, Ifnar1, Ifnb1, Ifng, Ifnl2, Il10, Il12a, Il12b, Il12rb1, Il13, Il15, Il18r1, Il2, Il21, Il23a, Il23r, Il27, Il27ra, Il2ra, Il33, Il4, Il4ra, Il5, Il6, Il7r, Irak3, Irf1, Irf3, Irf4, Irf5, Irf7, Itgal, Itgax, Jak3, Kit, Klrk1, Lef1, Lta, Masp1, Masp2, Mbl2, Mif, Mx1, Myd88, Nod2, Pirb, Pml, Pou2f2, Prf1, Prkcd, Ptger4, Ptpn6, Ptprc, Relb, Rorc, Serping1, Sh2d1a, Spn, Stat5a, Stat5b, Stat6, Sting1, Syk, Tap1, Tbk1, Tbx21, Tgfb1, Tgfb2, Tgfb3, Tlr2, Tlr3, Tlr4, Tlr8, Tlr9, Tnf, Tnfaip3, Tnfrsf4, Tnfsf18, Traf2, Traf3, Traf6, Trp53, Tyrobp, Xcl1 |
| **Immune response** | Aicda, Aire, Aqp4, B2m, Batf, Bcap31, Bcl2, Bcl3, Bcl6, Bst2, Btk, Btla, C1qa, C1qb, C1qbp, C1ra, C1s1, C2, C3, C4a, C4bp, C6, C8a, C8b, C8g, C9, Card9, Ccl11, Ccl12, Ccl2, Ccl20, Ccl22, Ccl24, Ccl25, Ccl3, Ccl4, Ccl5, Ccl6, Ccl7, Ccl8, Ccl9, Ccr2, Ccr4, Ccr5, Ccr6, Ccr7, Ccr8, Ccr9, Cd14, Cd19, Cd1d1, Cd226, Cd247, Cd24a, Cd274, Cd28, Cd36, Cd3e, Cd4, Cd40, Cd40lg, Cd44, Cd46, Cd55, Cd59b, Cd74, Cd79a, Cd79b, Cd86, Cd8a, Cfb, Cfd, Cfh, Cfi, Cfp, Ciita, Clec4e, Clec5a, Cr2, Csf1, Csf1r, Csf2, Ctla4, Ctsc, Ctsg, Cx3cl1, Cx3cr1, Cxcl1, Cxcl10, Cxcl11, Cxcl12, Cxcl13, Cxcl15, Cxcl16, Cxcl3, Cxcl9, Cybb, Ddx58, Defb1, Dpp4, Eomes, Fadd, Fas, Fasl, Fcamr, Fcer1a, Fcer1g, Fcgr1, Fcgr2b, Fcgr3, Fcgrt, Foxp3, Fyn, Gapdh, Gata3, Gpr183, Gzmb, H2-Aa, H2-Ab1, H2-DMa, H2-Eb1, H2-K1, H2-Q10, H60a, Hc, Hfe, Hlx, Icam1, Icosl, Ifi204, Ifih1, Ifit2, Ifitm1, Ifna1, Ifnb1, Ifng, Il10, Il12a, Il12b, Il12rb1, Il13, Il15, Il18, Il18r1, Il18rap, Il1a, Il1b, Il1r1, Il1rap, Il1rl1, Il1rl2, Il2, Il21, Il23a, Il23r, Il25, Il27, Il27ra, Il2ra, Il3, Il33, Il4, Il4ra, Il5, Il6, Il7, Il7r, Il9, Irak1, Irak3, Irak4, Irf1, Irf3, Irf4, Irf5, Irf7, Irf8, Irgm1, Itgal, Jak2, Jak3, Kit, Klrk1, Lck, Lef1, Lif, Lta, Ltb, Ly86, Ly96, Map4k2, Mapk1, Mapkapk2, Marco, Masp1, Masp2, Mbl2, Mif, Mr1, Mx1, Myd88, Naip5, Nfatc2, Nfkb2, Nfkbia, Nod2, Nos2, Notch1, Notch2, Pirb, Pou2f2, Pparg, Prf1, Prkcd, Ptafr, Ptger4, Ptpn22, Ptpn6, Ptprc, Rag1, Rela, Relb, Rorc, S100a8, S100a9, Serping1, Sh2d1a, Sigirr, Smad3, Spn, Stat1, Stat5a, Stat5b, Stat6, Sting1, Syk, Tap1, Tbk1, Tbx21, Tgfb1, Tgfb2, Tgfb3, Thy1, Tirap, Tlr1, Tlr2, Tlr3, Tlr4, Tlr5, Tlr8, Tlr9, Tnf, Tnfaip3, Tnfrsf11a, Tnfrsf13c, Tnfrsf1b, Tnfsf10, Tnfsf11, Tnfsf12, Tnfsf13b, Tnfsf14, Tnfsf15, Tnfsf18, Tnfsf8, Tollip, Traf2, Traf3, Traf6, Trp53, Tyrobp, Vegfa, Vtn, Xcl1, Zap70 |
| **Innate immune response** | Aqp4, Bst2, Btk, C1qa, C1qb, C1ra, C1s1, C2, C3, C4bp, C8a, C8b, C8g, C9, Card9, Ccl2, Ccl5, Cd14, Cd1d1, Cd226, Cd36, Cd55, Cd74, Cd86, Cfb, Cfd, Cfh, Cfi, Cfp, Ciita, Clec5a, Cr2, Csf1, Csf1r, Cxcl16, Cybb, Ddx58, Defb1, Fadd, Fcgr1, Gapdh, H2-Aa, H2-Ab1, H2-Eb1, H60a, Hc, Ifi204, Ifih1, Ifit2, Ifitm1, Il12a, Il12b, Il12rb1, Il18r1, Il18rap, Il1r1, Il1rap, Il1rl1, Il1rl2, Il21, Il23a, Il23r, Il27, Il4, Irak1, Irak3, Irak4, Irf1, Irf3, Irf5, Irf7, Irgm1, Jak2, Jak3, Klrk1, Ly86, Ly96, Map4k2, Mapkapk2, Marco, Masp1, Masp2, Mbl2, Mif, Mx1, Myd88, Naip5, Nfkbia, Nod2, Nos2, Pparg, Ptafr, Ptpn6, Rela, S100a8, S100a9, Serping1, Sh2d1a, Sigirr, Stat1, Stat5a, Stat5b, Sting1, Syk, Tap1, Tbk1, Tgfb1, Tirap, Tlr1, Tlr2, Tlr3, Tlr4, Tlr5, Tlr8, Tlr9, Tnfaip3, Tollip, Traf3, Xcl1 |
| **Lymphocyte chemotaxis** | Ccl2, Ccl3, Ccl4, Ccl5, Ccl7, Ccr2, Ccr7, Cx3cl1, Cxcl13, Cxcl16, Cxcr3, Xcl1 |
| **Mesenchymal cell differentiation** | Bcl2, Ctnnb1, Eomes, Hif1a, Lef1, Nfatc1, Notch1, Pax2, Plaur, Smad3, Tgfb1, Tgfb2, Tgfb3, Tgfbr1 |
| **Positive regulation of cell activation** | Aif1, Bcl2, Bcl6, Ccl2, Ccl5, Ccr2, Ccr7, Cd1d1, Cd226, Cd24a, Cd27, Cd274, Cd28, Cd3e, Cd4, Cd40, Cd40lg, Cd5, Cd59b, Cd74, Cd80, Cd81, Cd83, Cd86, Cdkn1a, Dpp4, Fcer1a, Fcer1g, Foxp3, Gata3, Gpr183, H2-Aa, H2-Ab1, H2-DMa, H60a, Hlx, Icosl, Ifng, Ikzf1, Il12a, Il12b, Il12rb1, Il13, Il15, Il15ra, Il18, Il1b, Il1rl1, Il1rl2, Il2, Il21, Il23a, Il2ra, Il2rg, Il33, Il4, Il4ra, Il5, Il6, Il6st, Il7, Il7r, Irf1, Itgal, Jak2, Jak3, Lck, Mif, Myd88, Nfatc2, Nod2, Pdcd1lg2, Prdm1, Ptprc, Spn, Stat5a, Stat5b, Stat6, Syk, Tbx21, Tgfb1, Tgfbr2, Thy1, Tirap, Tlr4, Tnfrsf13c, Tnfrsf4, Tnfsf11, Tnfsf13b, Tnfsf14, Traf6, Vcam1, Xcl1, Zap70 |
| **Positive regulation of neurogenesis** | Ctnnb1, Cxcl12, Cxcr4, Grm5, Hif1a, Il1b, Il6st, Kit, Lif, Mapt, Mif, Notch1, Olig2, Pparg, Ptk2, Rela, Vegfa |
| **Response to biotic stimulus** | Aicda, B2m, Batf, Batf3, Bcl2, Bcl3, Bst2, Btk, C1qbp, Camp, Card9, Casp1, Ccl2, Ccl3, Ccl5, Ccr5, Ccr7, Cd14, Cd160, Cd226, Cd24a, Cd36, Cd4, Cd40, Cd40lg, Cd86, Cd8a, Cebpb, Chuk, Clec5a, Ctsg, Cxcl10, Cxcl13, Cxcl15, Cxcl9, Ddx58, Defb1, Defb14, Fadd, Fcer1g, Fcgr1, Fcgr2b, Fcgr4, H2-Eb1, H2-K1, H2-Q10, H60a, Hamp, Ifi204, Ifih1, Ifit2, Ifitm1, Ifna1, Ifna2, Ifnar1, Ifnb1, Ifng, Ifnl2, Il10, Il12a, Il12b, Il12rb1, Il12rb2, Il15, Il18, Il1b, Il23a, Il23r, Il25, Il27ra, Il2ra, Il4, Il4ra, Il6, Irak1, Irak2, Irak3, Irf1, Irf3, Irf4, Irf5, Irf8, Itgax, Itln1, Jak2, Klra8, Klrk1, Litaf, Lta, Ly96, Mapk1, Mapk14, Mapkapk2, Mbl2, Mif, Mr1, Mx1, Myd88, Naip5, Nfkbia, Nod2, Nos2, Nos3, Pml, Prdm1, Prf1, Prkcd, Ptafr, Ptger4, Ptgs2, Ptprc, Rela, Smad3, Spn, Stat1, Stat5b, Sting1, Syk, Tbk1, Tgfb1, Tirap, Tlr1, Tlr2, Tlr3, Tlr4, Tlr8, Tlr9, Tnf, Tnfaip3, Tnfrsf11a, Tnfrsf14, Tnfrsf1a, Tnfrsf1b, Tnfsf8, Traf3, Traf6, Trp53, Xcl1 |
| **Response to interferon-gamma** | Aqp4, Bst2, Ccl2, Ccl5, Ciita, Cxcl16, Gapdh, H2-Aa, H2-Ab1, H2-Eb1, Ifitm1, Il12b, Il12rb1, Il23r, Irf1, Jak2, Nos2, Pparg, Stat1 |
| **Response to virus** | Batf3, Bcl2, Bcl3, Bst2, C1qbp, Card9, Ccl5, Cd40, Cd86, Cd8a, Clec5a, Cxcl10, Cxcl9, Ddx58, Fadd, Ifi204, Ifih1, Ifit2, Ifitm1, Ifna1, Ifna2, Ifnar1, Ifnb1, Ifng, Ifnl2, Il12b, Il12rb1, Il15, Il23a, Il23r, Il2ra, Il6, Irak3, Irf1, Irf3, Irf5, Itgax, Klra8, Mx1, Myd88, Pml, Prf1, Ptprc, Spn, Sting1, Tbk1, Tlr3, Tlr8, Tlr9, Tnf, Traf3, Xcl1 |
| **Tissue remodeling** | Ahr, Bax, Cd24a, Csf1r, Ctnnb1, Ctss, Hif1a, Il18, Il23a, Il7, Itga4, Lif, Nos2, Nos3, Notch2, Nox4, Src, Stat5a, Syk, Tfrc, Tgfb2, Tnfsf11, Traf6, Trp53, Vegfa |

**Table S1. The list of genes included in upregulated or downregulated GO pathways in GAGE analysis**
