# Supplementary figures and images for: Characterization of the therapeutic effect of antibodies targeting the Ebola glycoprotein using a novel BSL2-compliant rVSVΔG-EBOV-GP infection model
Source: Emerg Microbes Infect. 2021 Nov 10;10(1):2076–89. doi: 10.1080/22221751.2021.1997075 (PMC8583756; doi:10.1080/22221751.2021.1997075)

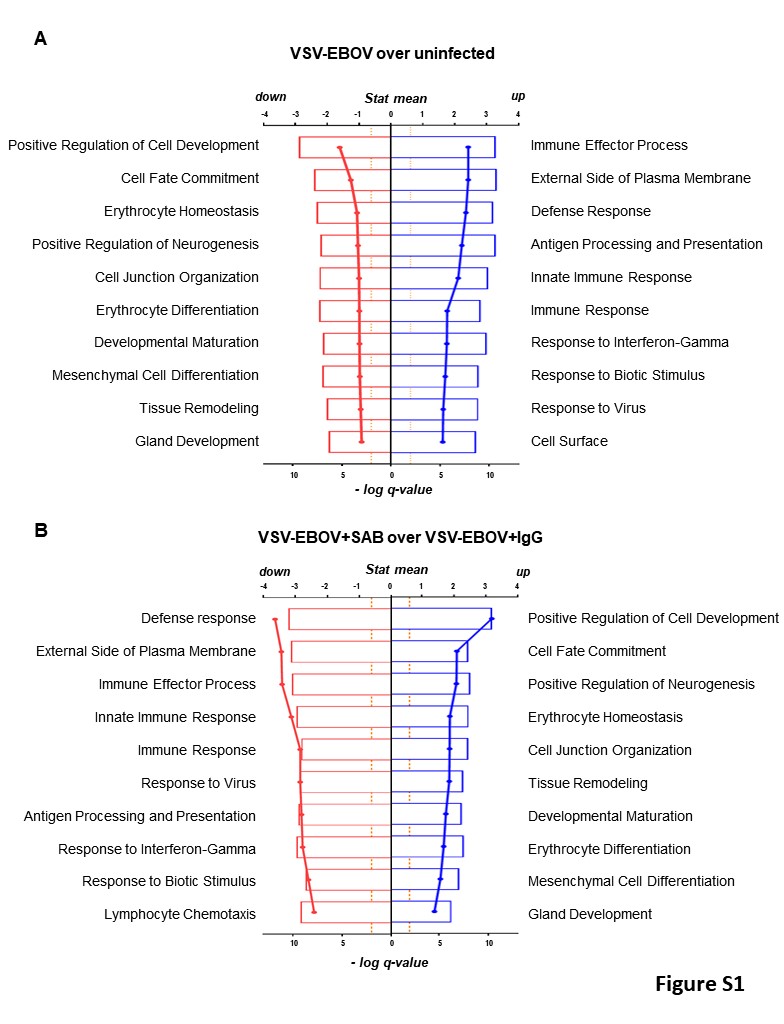

Supplement: Supplemental Material [file TEMI_A_1997075_SM3280.zip › TEMI 1997075_Supplmentary Files/Figure S1.JPG]

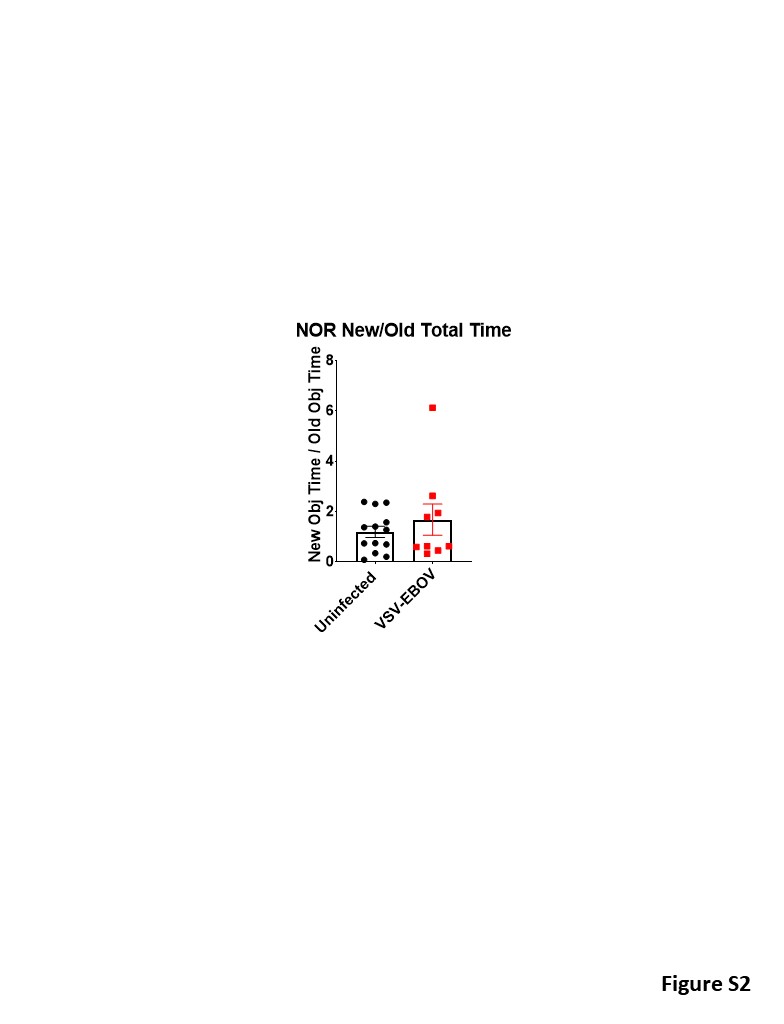

Supplement: Supplemental Material [file TEMI_A_1997075_SM3280.zip › TEMI 1997075_Supplmentary Files/Figure S2.JPG]

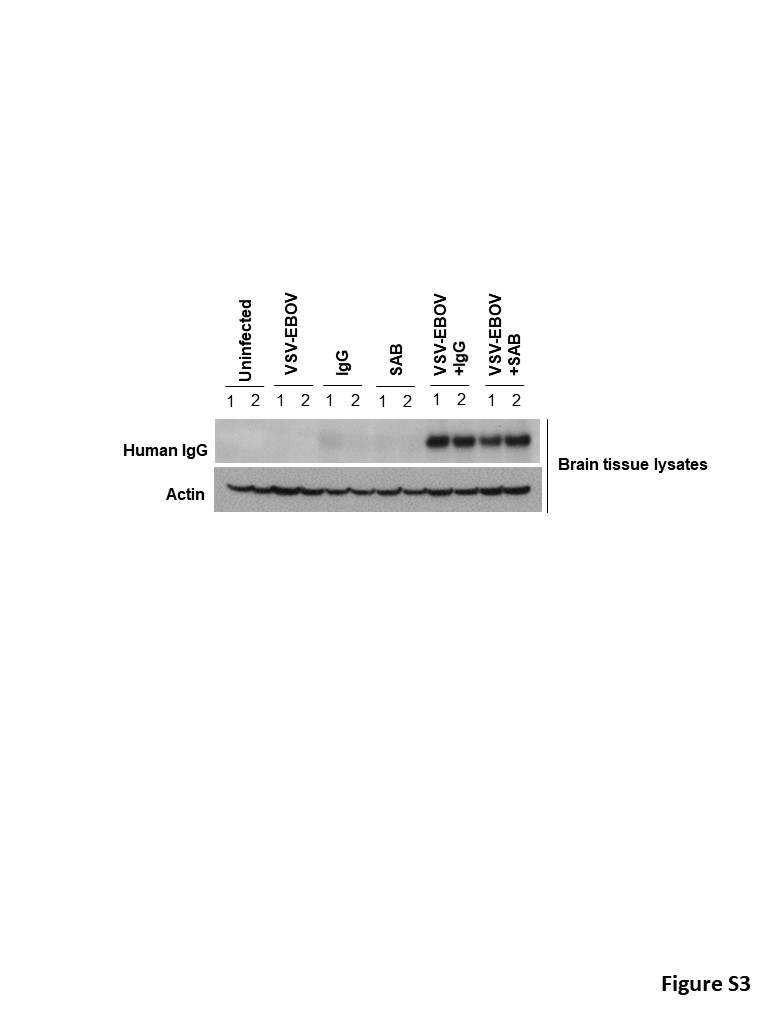

Supplement: Supplemental Material [file TEMI_A_1997075_SM3280.zip › TEMI 1997075_Supplmentary Files/Figure S3.JPG]
